# Internal Abiotic Components That Influence the Development of Biocorrosion on ETICS Plasters

**DOI:** 10.3390/ma15010127

**Published:** 2021-12-24

**Authors:** Monika Dybowska-Józefiak, Maria Wesołowska

**Affiliations:** Faculty of Civil and Environmental Engineering, and Architecture, Bydgoszcz University of Science and Technology, S. Kaliskiego 7, 85-796 Bydgoszcz, Poland; monika.dybowska@pbs.edu.pl

**Keywords:** biocorrosion, ETICS system, facade surface

## Abstract

Basic factors affecting the appearance of algae discoloration on the surface of the system are recognized effects of the external environment (external temperature and humidity, short- and long-term radiation, precipitation, wind and air pollution). Internal factors are often neglected by international technical documents on the evaluation of the effectiveness of resistance to biocorrosion of the External Thermal Insulation Composite System (ETICS). Based on literature data and in situ research, the basic internal factors responsible for the occurrence or intensification of the biocorrosion process were systematized. Internal factors were divided into two groups: (1) plaster properties and (2) solutions for material layouts and building details. The results of research on these factors indicate that they directly or indirectly influence the humidity condition of plaster and biocorrosion development is a consequence of this state. The opposite issue, the influence of biocorrosion on plaster properties, is analyzed only in patrial way.

## 1. Introduction

ETICS stands for External Thermal Insulation Composite System (this building system is often called EIFS in North America) [1]. The system was developed in Germany in 1950 and 1960 [2], where many brick houses required additional thermal insulation. A layer of thermal insulation was then placed on the outside of the original facade and plastered over. This solution also became popular in Scandinavia during the energy crisis of the 70s of the XX century [3]. Currently, it is used throughout Europe, the United States and Canada. According to Duarte [4], it is now particularly popular in Germany, Poland and the Czech Republic. The Polish ETICS market, estimated at approximately 40 mm^2^/year, is one of the leading markets in Europe [5]. This system is used in both new and renovated buildings.

The insulation of external walls of buildings with this system was performed according to the technical specification of EAD 040083-00-0404 [6], which superseded the previously used specification of EOTA ETAG 004 [7]. Based on the fastening method of the thermal insulation, there are four types of ETICS system:

– Bonded with a minimum 20% of adhesive surface;
– Bonded with additional mechanical fastening and a minimum 20% of adhesive surface;
– Mechanically fastened with an additional bond with a minimum 20% of adhesive surface;
– Mechanically fastened with adhesive surface smaller than 20%.

The basic components of the system are the following:

1. Adhesive mortar for fixing thermo-insulated panels to the substrate;
2. Thermo-insulated panels (thermal insulation products that can be evaluated according to the methods listed in the EAD) [6];
3. Mechanical connectors;
4. Finishing layer consisting of the following components:
  – Mortar or adhesive compound for embedding render mesh;
  – iberglass mesh or metal mesh;
  – Optionally:
    ● Primer for external coating;
    ● Thin layer mortar or plaster coating with differentiated composition and texture;
    ● Exterior wall paint with a primer that matches paint type.

**Figure 1:**
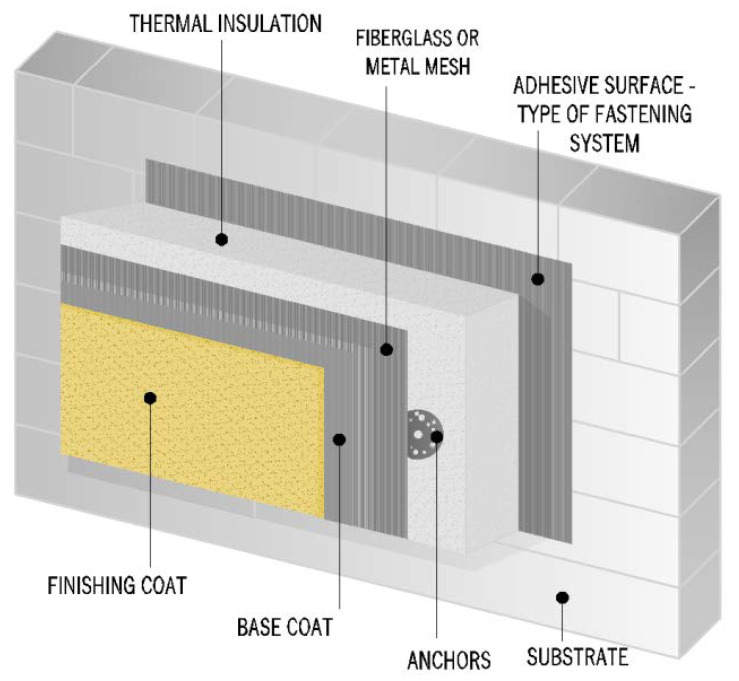
Schematic example of ETICS available in the European market.

The insulation substrate can be a masonry wall (brick, block) or concrete wall (monolithic or prefabricated) with or without plaster systems. Figure 1 shows the example application scheme of the ETICS system according to European requirements.

**Figure 2:**
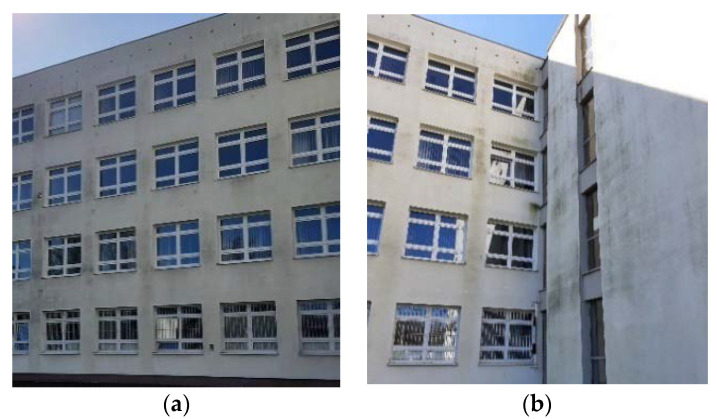
Changes on the exterior wall finish surface (author’s archives) (**a**) Uniformly distributed growth on the exterior wall finish, (**b**) Local contamination concentrations.

Beneficial long-term properties combined with excellent protection against blowing rain and high thermal insulation quality are the reasons why ETICS has become so popular in Central Europe. The multilayer exterior coating was evaluated as a less vulnerable solution in comparison with traditional plaster coatings [2]. However, earlier ETICS applications revealed particularly poor resistance to impact and biological infestation of plasters [8]. In the light of practical experience and theoretical studies, the durability of an insulation system depends largely on the properties of individual components, such as Styrofoam boards, priming layer, adhesive mortar and plaster layers, as well as on the way they are combined with one another. The weakest of these is exterior wall finish plaster, which causes defects such as cracking, flaking or peeling off the surface and, sometimes, some fragments falling off. Changes to the exterior wall finish surface due to microbial growth are generally accepted as ‘patina’ as long as they are uniformly distributed, while local contamination or algae concentrations are often evaluated as ‘visually adverse’ [2]—Figure 2.

The basic properties of the ETICS system are defined in the technical specification EAD 040083-00-0404 [6]. Apart from the requirements for fire, thermal and acoustic protection related to the insulation system, this document also defines a group of physical features concerning the external coating: substance leaching, water absorption, water tightness, frost resistance, impact resistance, water vapor permeability. However, this document does not contain direct requirements that relate to the protection against biocorrosion.

Infestation of ETICS plasters with biological corrosion is a result of biotic (biological) and abiotic (physical and chemical) factors. Organisms functioning on the exterior walls have different requirements in terms of these factors. Their interaction determines whether an organism can thrive in a given environment [9].

Abiotic factors can be classified into two main groups:

- Group 1—effect of internal factors such as plaster properties (thermal capacity, plaster composition, surface structure) and solutions for material layouts and building details;
- Group 2—effects of the external environment in the form of outside air temperature and humidity, short wave radiation, precipitation, wind and air pollution.

The effect of these groups is mainly the moisture condition of the plaster coatings.

Taking the above into account, the aim of this paper is to analyze the current state of knowledge concerning experimental and simulation studies in the field of susceptibility of the ETICS plasters to environmental impact. Result systematization of this research will have not only a cognitive value but also a great practical significance. It will provide an answer to the question, how abiotic factors influence the growth and biological activity of organisms living in extreme environments, that is, the building exterior wall finish.

## 2. Plaster Properties

### 2.1. Plaster Thermal Capacity

**Figure 3:**
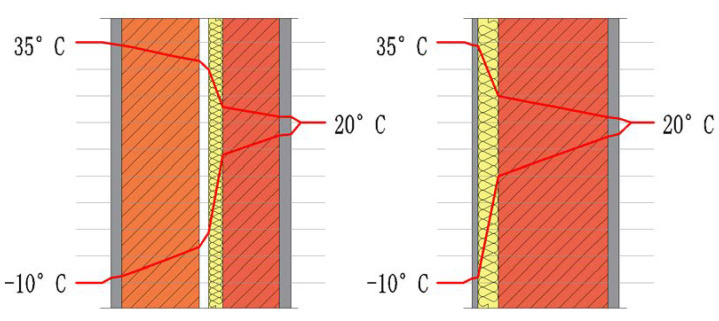
Temperature variation in exterior walls (author’s archives based on [8]).

The thermal capacity of thin-coat plaster is low and does not provide sufficient thermal inertia to avoid lowering the surface temperature at night. Heat radiates much faster from the ETICS system external layer than from a traditional envelope component; therefore, the surface temperature drops significantly [10]. Daytime surface temperature fluctuations on thin-clad exterior wall finishes are much higher than in the case of heavy-clad ones as the Sun heats the surface with short-wave radiation. However, light exterior wall finishes become cooler at night compared with heavy ones due to their lower thermal inertia. Due to higher thermal inertia, heavy exterior wall finishes can store heat from day to night. In addition, due to the long-wave radiation from the sky, the light exterior wall finish can reach temperatures even below the ambient temperature. This happens especially at night with a clear cold sky, which is typical of the transitional seasons. This effect never occurs in the case of heavy exterior wall finishes due to the higher thermal inertia of this structure [11]. Exterior wall finish surfaces with low thermal inertia remain wet longer due to condensation than heavy exterior wall finishes because of their lower temperature and longer drying time. If the drying process is not fast enough, the surface moisture remains high for a long time and increases the risk of microbiological growth [12]. The most important parameters in the drying process are the absorption of short-wave radiation, solar radiation and orientation [8]. The insulation lies on the outside of the wall resulting in a higher thermal mass on the inside. This increases the thermal comfort during the cold season, as solar gains also increase, and during the warm season, delaying and moderating fluctuations of the heat flow that helps maintain the building temperature. There is also an increase in the facades’ durability as the masonry is better protected from the climate loads (thermal gradient—Figure 3, wind-driven rain, etc.).

One of the future trends in building materials development is activation of opaque facades to achieve hybrid materials with changeable conductivity and isothermal storage of heat. Simo Ilomets et al. propose a method for developing an Energy Activated External Thermal Insulation Composite System (En-ActivETICS) for smart building envelopes. The idea how to develop a smart self-regulated energy active building envelope component is based on the combination of ETICS with a phase change material (PCM) and flexible photovoltaic (FPV) cells. A PCM is a substance that has the ability to store and release a large amount of energy during the phase transition. The process is semi-isothermal which results in periodical stabilization of the medium at a designed temperature level [13].

### 2.2. Plaster Composition

There are two types of coating mortars used in recent years in the construction industry: two- or three-layer cement-sand grout mixed on site (traditional plasters) and factory-made mortars made of cement, sand, admixtures and additives in one layer (ready-mixed plasters) [14], where the finishing layer is the plaster itself (‘single-layer mortars’) or other materials (e.g., paint). The use of factory-made mortars began in the 1990s in Portugal [15] and is steadily gaining momentum.

From a functional point of view, external mortars contribute significantly to the wall structure tightness (primary function). Moreover, they condition its aesthetics (secondary function) and have a decisive influence on its durability [14].

The protective function in ETICS systems is mainly achieved with structural plasters. The use of structural plasters in ETICS systems should be based on the system structure and the results of thermal and moisture calculations (elimination of interlayer condensation) but also on the place of installation and analysis of other characteristics and properties. They should have the following characteristics: low water absorption, diffusivity, resistance to dirt, resistance to biological infestation, UV resistance [15].

Basic textured plasters include:

- Mineral-based plasters (mineral polymer): It is a dry mixture of cement, lime, aggregate and organic additives. They are durable, vapor permeable, low-absorbent and difficult to clean. They are resistant to weather conditions but sensitive to execution conditions [16]. Available in a limited range of colors, usually soft tone colors;
- Acrylic plasters that feature high elasticity at low vapor permeability and good adhesion to the substrate. They are resistant to adverse execution conditions [16]. Acrylic plasters are products based on synthetic (acrylic) resins with various additives. Offered in a wide range of colors, they are non-absorbent and water resistant. Their main disadvantage, apart from low vapor permeability, is their susceptibility to contamination due to electrostatic properties and near neutral pH. This is why they are sometimes modified, e.g., with Teflon, which reduces their susceptibility to contamination and makes them easier to clean. They contain organic compounds which constitute a good substrate for algae, mold and other microorganisms;
- Silicate plasters: Their base is potassium water glass with the addition of polymer resin dispersion and modifiers that reduce absorption. They are vapor permeable and rain resistant. They are resistant to atmospheric conditions, easy to clean due to their strong alkaline pH value and more durable than mineral or acrylic plasters [16]. Available in a limited color palette due to alkalinity;
- Silicone plasters which combine the advantages of mineral and resin plasters. They are polymer products (based on synthetic resins), just like acrylic plasters, but they are significantly different from them. First of all, they are vapor permeable, which means they can also be used on mineral wool. They feature high flexibility and adhesion. Their surface is not wettable with water, so precipitation washes away contamination from the exterior wall finish. They are very durable, and resistant to many factors.

Exterior wall finish coatings are thoroughly colored or painted with exterior wall coatings that are based on acrylic, silicate or silicone water dispersions [17]. Acrylic and silicone plasters, thanks to their hydrophobic properties, do not attract water molecules and make it difficult for contaminants to settle. On the other hand, hydrophilic plasters include mineral plasters and standard silicate plasters that are highly absorbent. Silicate and silicone plasters are, in fact, silicate plasters that are modified with a special type of silicone resin featuring additional binding properties. This way their absorbability and sensitivity to external conditions are reduced.

In fact, the potassium silicates of the coating (both the key-coat and the paint) physic chemically bonds (silification) to the calcium and other inorganic mineral matter of the base coat [18]. Thus, the base coat and the finishing coat end up as a single compound and/or with a strong adhesion, providing improved water repellency properties to the system ETICS.

Polymer coating, on the other hand, physically adheres to the substrate and forms an interface; in fact, the formation of acrylate-siloxane copolymer (hybrid polyacrylic–polsiloxane structure) can provide water repellency (due to the methyl groups), antimicrobial growth inhibition and enhanced adhesion properties [19,20,21]. Thus, the acrylic film is not chemically bonded to the base coat, justifying its slightly higher water absorption when compared to that of a coat with silicate-based paint.

Research concerning the algae infestation on exterior walls in Polish climatic conditions show that the highest algae infestation can be found on mineral plasters (54%). Other types of plasters reach a few to several percent: 13% in the case of polymer plasters, 10% for silicone plasters, 5% for silicate plasters and 18% for other types [22].

### 2.3. Plaster pH

The substrate pH value is also an important parameter. Most microorganisms thrive best at a pH close to neutral, that is, in the pH range of 5–8. However, many of them, such as mold or yeast, thrive at a pH range of 4.5–5 [23].

It is assumed that a higher pH, specific for the surface of mineral and silicate plasters, creates a greater barrier for the colonization of their surface by microorganisms. Therefore, they should be more resistant to growth than polymer or silicone plasters in a pH range of 7–9 [24].

The plaster pH is higher than 10.5 (strongly alkaline), which effectively prevents the development of microorganisms (for which the beneficial pH value is between 7 and 8.5). Fresh exterior wall finishes in the first years of operation (approximately 2–3 years, depending on the environment) feature natural protection due to their pH value. However, this effect diminishes over the years. Plaster durability is estimated at 5 years. After this time, renovation measures such as cleaning and painting may be necessary [25]. Measurements of the pH level carried out by Horbik [26] for different surfaces contaminated with fungi (granite, ceramics, concrete and cement-lime plaster) confirmed the pH decrease in the substrate with time (Table 1).

Over time, a decrease in pH value is observed, which initially (during the first month) is relatively small. Another five months of fungal activity results in a further, more significant drop in pH value by 2.5 in the case of cement-lime plaster. The fungus-secreted substances react with phases that contain calcium and form soluble compounds that are removed from materials. Etching process of these materials and their slow decomposition occurs [26]. As a result, building materials change their pH over time, making them more susceptible to rapid infestation with algae and fungal spores.

### 2.4. Surface Structure

**Figure 4:**
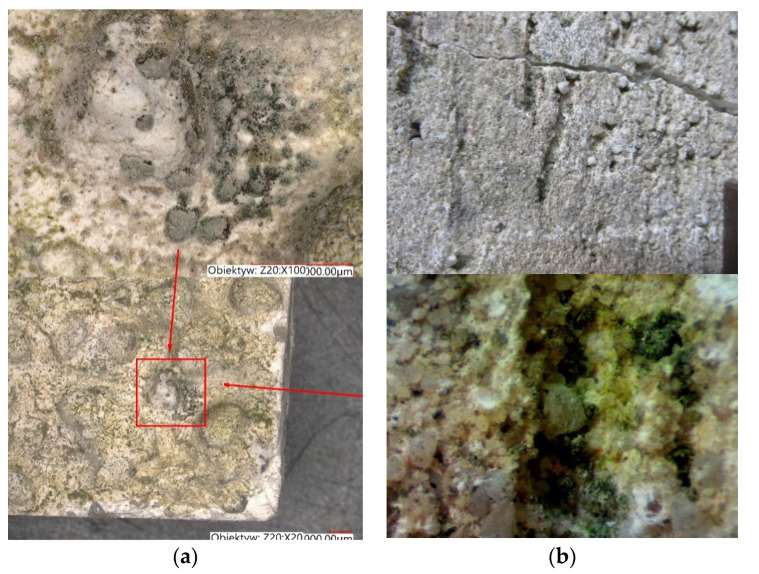
Development of biocorrosion on different textures after 5 years of operation (magnification: 20x and 100x) (author’s archives). (**a**) Dashed plaster. (**b**) Filled plaster.

Over the past thirty years, many studies have investigated the bioreceptivity of building materials. Experimental tests were performed on materials using accelerated biological growth methods. Their goal was to reduce the study duration which, under real conditions, usually takes many years [27]. Experimental parameters were therefore optimized to prioritize microorganism growth (Barberousse [28]). According to these studies, the colonization of building materials by microorganisms is promoted with surface roughness. In fact, roughness provides many bumps, and thus increases the physical anchor points for such microorganisms (Tran [27]). The effect of roughness on microorganisms was confirmed with observations of buildings in real conditions [28,29,30,31,32,33,34]. Rough surfaces provide ideal breeding grounds for microorganisms, as coarser, more pronounced grain structures tend to retain contamination in the inter-grain area much longer. This is due to the fact that it is more difficult to wash out the contamination with rain. Textures with pronounced, protruding or concave shapes, such as filled plaster or modeled plaster, are particularly susceptible. Their character, i.e., horizontal scratches/dents, etc., facilitates the accumulation of contamination and therefore also acts as a nourishing substance for microorganisms [23,25,35,36]. Examples of biocorrosion stages on different textures are shown in Figure 4.

Roughness promotes physical adhesion of microorganisms that are dispersed with water flow or wind. Consequently, the rougher the surface is, the earlier the biological contamination process begins and the faster the colonization of microorganisms occurs. Surface roughness can be evaluated using an Elcometer 223 surface profile gauge. This device can measure the peak-to-valley of a surface up to 2 mm, with a resolution of 0.001 mm. The systems, which have an acrylic paint in the finishing coat, present higher values of surface roughness [18].

Experiments conducted by Tran et al. [27,37] with accelerated contamination deposition confirm the roughness effect. Applied three degrees of roughness produced a differential effect on colonization rate. Three levels of roughness were obtained regardless of curing process and w/c ratio. The smoothest surface (R1), without protrusions, was obtained with the finishing method according to this rule. Rougher surfaces were obtained with scratching the sample surface with sponges. The roughest surface (R3) showed both large protrusions and high roughness. Moreover, a clear difference was observed between the highest roughness (R3) and the intermediate roughness (R2). Algae growth formed streaks on the sample surfaces with the lowest roughness (R1 and R2) due to slurry flow. This form of contamination was usually observed on the exterior wall finish of buildings. In case of mortars with the highest roughness (R3), contamination occurred due to the formation of surface irregularities that conducted the flow of algal slurry. This study investigates the influence of internal parameters (porosity, roughness and carbonation) of masonry mortars on biological growth with photosynthetic organisms. The research was conducted using both laboratory-accelerated and in situ testing methods. The effect of roughness was proven in both test scales. Rough surface increases biological adhesion. The differentiation of roughness classes was better in accelerated tests than in field-scale tests. This result can be explained by the fact that the accelerated test is performed in a closed circuit with intensive inoculation, whereas the in situ test relies on natural inoculation [27].

### 2.5. Plaster Porosity

Water absorption and retention in materials are controlled by total porosity and pore size distribution (Tran at al. [27]). The effect of porosity and its structure on the physical properties of building materials was confirmed by Neville [38], Fagerlund [39] and other researchers [40,41,42,43,44,45,46] who conducted studies on concrete, mortar, cement paste, ceramic stone, gravel and other materials. It is generally known that smaller pore volume has good influence on the properties of cement materials [41,43,44,46]. The hardening process, which can take more than a year, can also increase the number of micropores and mesopores of less than 100 nm in size and reduce the number of capillary pores, which improves the material tightness. This promotes less penetration of moisture and SO_2_, NO_x_ aggressive gases [47] into the microstructure of plasters, and thus improves the material durability. For this reason, the types and size of pores are quite important, especially open pores that allow penetration of liquids and gases. For example, according to Neville [38], a large number of pores less than 5 nm in size hinders water penetration. In pore sizes of 5–100 nm in size, water can flow due to diffusion. In larger pore sizes of 0.1 µm in size, water can flow due to capillary adsorption. In addition, at low temperatures in pores of 0.1–1.0 µm size, which are crucial, freezing water causes the largest damage in material [39].

The porosity and pore structure variations during aging processes can be useful in terms of aging and durability studies, especially when there is no method for the evaluation of durability of external plasters. Bochen conducted a study of the variability of the porosity variations of mineral facade plasters subjected to the natural and accelerated aging tests, as recommended by the durability procedures [48]. For the aging tests, four types of mortars mixtures were prepared. Two ordinary mortars with cement and cement–lime as binder, and two thin-layer types made of modified mixtures with the additions cement–lime Y (Ytong system) and cement E (Euromix system). Plasters were prepared to achieve class M3. Porosity changes are presented in Table 2.

## 3. Solutions for Material Layouts and Building Details

### 3.1. Thermal Requirements for Envelope Components

The requirements for thermal insulation of external walls have increased significantly over the last fifty years. Eventually, in 2021, the acceptable coefficient values are about six times lower than those originally defined [49]. Ensuring proper wall insulation requires to utilize multilayer systems with appropriate thicknesses of thermal insulation. As a consequence, the temperatures on the external wall surface are becoming lower and lower, which causes the prolongation of the condensation period during transitional seasons of the year and generates the so-called periods of summer condensation [50].

**Figure 5:**
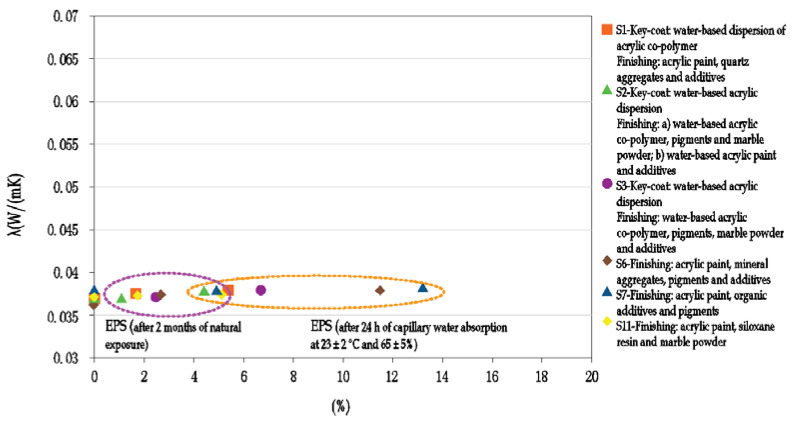
Thermal conductivity of the thermal insulation’s material as a function of moisture content (author’s archives based on [20]).

The effect of moisture variation on the thermal conductivity of the ETICS insulation layer was investigated in J.L. Parracha et al. An increase in the thermal conductivity with moisture content was obtained for all the tested systems, regardless of the thermal insulation material used (EPS- Expanded polystyrene, ICB- Expanded cork agglomerate or MW- Mineral wool) - Figure 5 [20].

Even if moisture achieves the insulating layer, it induces no significant variation on its thermal capacity. This can be explained by the internal closed pore structure of EPS as well as by the high resistance to water penetration (both in the liquid and vapor phase) obtained for the EPS systems. However, results confirm that the thermal conductivity is always affected by moisture content [20].

### 3.2. Plaster Color Scheme

Surface color directly affects the absorption coefficient of short-wave radiation. Research conducted by Dylla [51] and Rokiel [15] shows that white exterior wall finish can have an absorption coefficient of 0.3 while dark surfaces have values close to 1.0. The result is a warming up of the dark colored exterior walls. Thermal stresses that appear in the insulation layers are closely related to the insulation of the thin coating from the substrate with thermal insulation layer. In conditions of intensive sun exposure, plaster layers, deprived of transferring heat deep into the envelope component, become considerably heated. Intensive stresses occur in the plaster layers of small thickness and volume (5–7 mm), causing cyclic material deformation, which usually leads to microcracks and, in extreme cases, even to damaging the thermal insulation layer.

**Figure 6:**
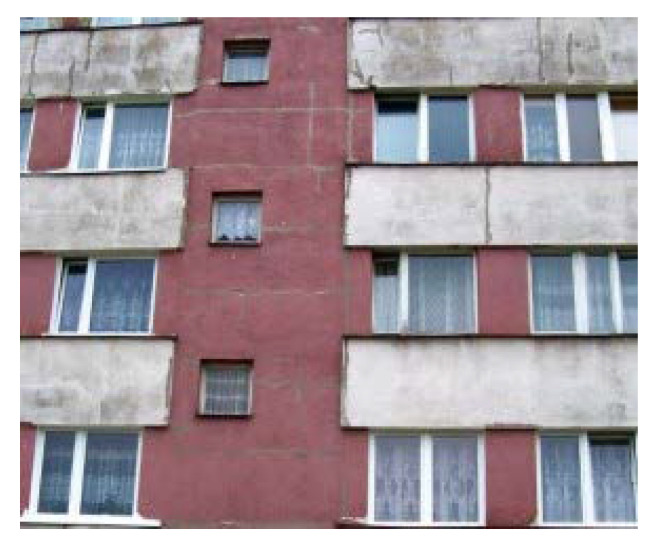
Exterior wall finish biocorrosion with different colors (author’s archives).

On the other hand, faster heating and heat accumulation of the darker parts of the buildings contribute to lower tendency of microbial growth (Figure 6).

**Figure 7:**
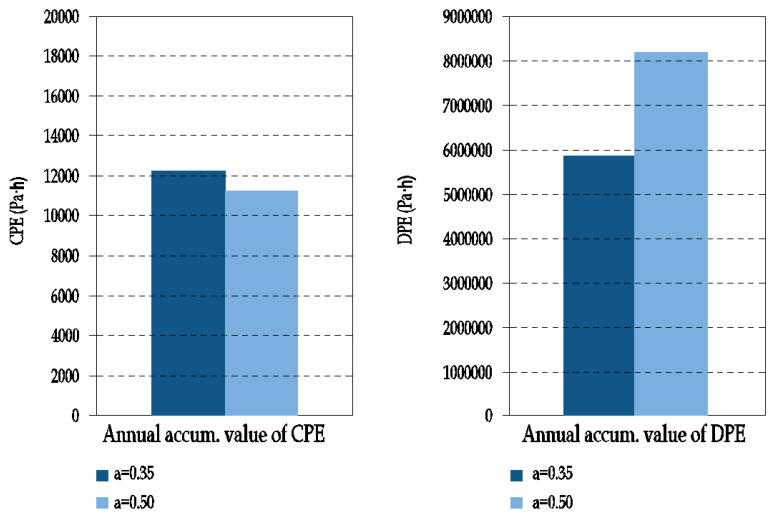
Properties of the exterior layer: absorbance to solar radiation (author’s archives based on [8]).

Due to the climatic conditions of Poland, the outside air temperature in summer is 19.1 °C [52]. As a result of absorption of short-wave radiation, the surface temperature of the plaster will be higher. According to the research conducted by Rokiel [50], the temperature of black textured plaster insulation, exposed to sunlight, exceeds +52 °C, assuming that the outside air temperature in the shade is +25.2 °C. The consequence is the change of temperature distribution in the whole envelope component (Figure 4), which promotes drying. However, at the boundary of various colors, it may cause tensile stress in the plaster and as a result, its cracking. In extreme cases, it may even damage thermal insulation boards. The short-wave absorption coefficient affects the solar radiation absorbed by the wall during the day and changes the surface temperature. Its effect on surface temperature is quite significant during the day but at night, due to low heat capacity of the plaster layer, the stored heat is quickly lost and the temperature rises only slightly. A similar study, conducted by Fraunhofer-Institute for Building Physics (Figure 7), came to the same conclusions for both emissivity and absorption of short-wave radiation, taking into account the Holzkirchen climate [53,54,55].

### 3.3. Exterior Coating Material Systems

In the case of a two-layer external wall, the selection of the external coating material system depends on the thermal insulation material that was used. Wesołowska, in her study [56], proposed a selection condition using the basic performance properties of thermal insulation materials and coatings:

S_d,izol_ ≥ S_d, plaster_ ≥ ew.S_d,paint coat_, (1)

where:

S_d,insulation_ = μ∙d, [m] (2)

S_d, insulation_—water vapor diffusion, equivalent air layer thickness for a thermal insulation.
μ—water vapor resistance factor for a given material.
d—material thickness.
S_d, plaster_—water vapor diffusion, equivalent air layer thickness according to Table 3.
S_d, paint coa_—water vapor diffusion, equivalent air layer thickness according to the declaration of performance.

In case of diffusively open thermo-insulated materials (e.g., mineral wool), only Group I coatings should be used. For other materials, all coating classes are permitted, provided that the requirement (1) is met.

Predicting the risk of surface colonization with biological agents involves the correlation between the wall surface absorption coefficient (w) and the diffusion resistance (Sd). Both values should be low and should not exceed [4,51]:

w < 0.5 kg/(m^2^·h^½^) and S_d_ < 2.0 m, (3)

and

(w)·(S_d_) < 0.2 kg/(m^2^·h^½^). (4)

In all envelope components, regardless of the insulation material type and the applied layer arrangement, discontinuous preparation of the thermal insulation layer or leaving gaps at the envelope component joints may cause moisture condensation and accumulation and destruction of the insulation material and envelope components [57].

**Figure 8:**
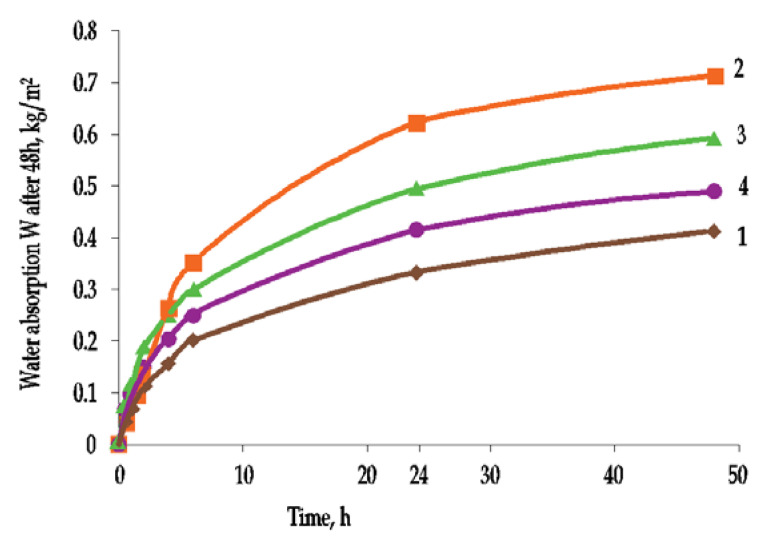
Surface water absorption of ETICS samples: (1) from acrylic plaster, (2) from silicate plaster, (3) from mineral plaster and (4) from silicone plaster (author’s archives based on [28]).

**Figure 9:**
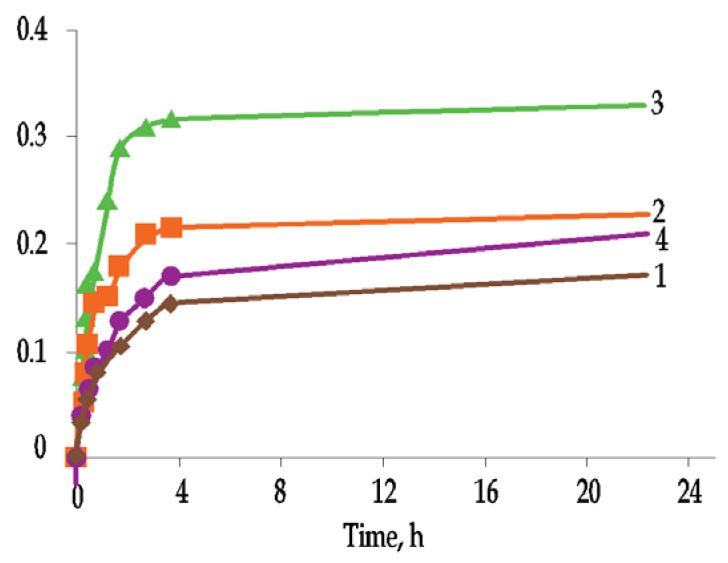
Surface water absorption of exterior plaster samples on plastic strip: (1) acrylic plaster, (2) silicate plaster, (3) mineral plaster and (4) silicone plaster (author’s archives based on [28]).

The layered arrangement of the ETIC system will also cause water absorption in the thermal insulation material. This effect was described in detail by Tran et al. [27]. The results of their research (Figure 8) showed that, after immersing ETICS samples in water, the intensity of water absorption decreases after about 24 h. The same water absorption test was carried out with external plaster coatings on plastic tape (Figure 9). The rate of water absorption decreased after 4 h. After that time, water continues to be absorbed but very slowly.

These studies clearly indicate that the water absorption of the entire ETICS system is more intense and achieves higher values than the water absorption of the ETICS exterior plaster on hydrophobic solid plastic strip. For accelerated aging, it is important to analyze the physical changes of the entire ETIC system, though the maximum negative impact of water occurs in the external plaster layer. These studies also confirm that for the entire ETICS plaster to be saturated with water, the rain should last at least 4 h. Analysis of water absorption of ETICS systems showed that silicate plaster absorbs the most water of all plasters (0.62 kg/m^2^ per 24 h), while acrylic plaster absorbs less (0.33 kg/m^2^ per 24 h). However, according to the indicators of water absorption from the external plaster layer, the maximum water absorption rate is achieved with mineral plasters (0.33 kg/m^2^ per 24 h), and the minimum water absorption rate is achieved with the acrylic layer (0.17 kg/m^2^ per 24 h). This means that the initial rate of water absorption depends not only on the type of external layer but also on the deeper layers of the ETICS system, i.e., reinforcement layer [27].

### 3.4. Structural Solutions of ETICS System

**Figure 10:**
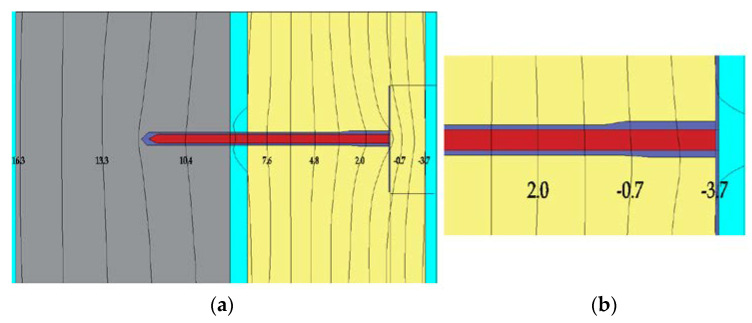
Compared distribution of isotherms for two mounting types of the connector: (**a**) mounting with Styrofoam, (**b**) mounting without Styrofoam plug (author’s archives based on [59]).

**Figure 11:**
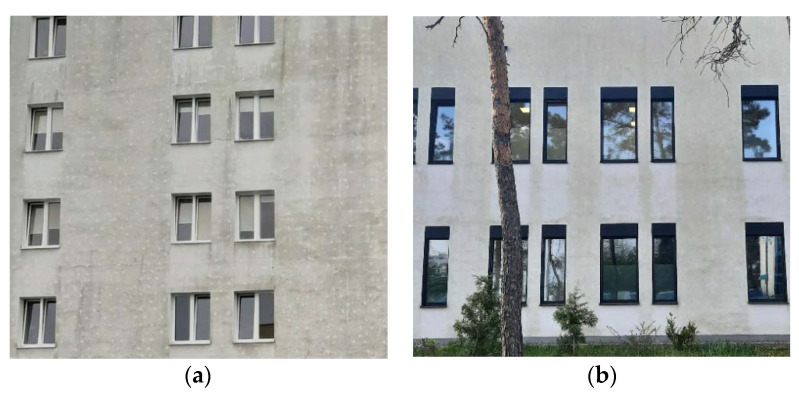
Specific biocorrosion development on exterior wall finish of the same building in operation for 10 years (photo (**a**)) and for 5 years (photo (**b**)). (**a**) Anchors that completely penetrate the thermal insulation material (author’s archives). (**b**) Anchors embedded into the insulation material (author’s archives).

The type and method of installation of the system mechanical connectors affect the possibility of temperature differences on the envelope component surface. The connectors are the so-called point thermal bridges, the influence of which should be treated as a thermal disturbance [58]. There is an increased heat loss through the thermal bridge, which is directly proportional to the size of the thermal bridge. The connectors used for fixing thermal insulation in ETICS systems have a specific point thermal transmittance coefficient, described as χ (W/K), which is specified and specified for a given fastener, as is the plate stiffness and the specific load capacity. During thermal imaging studies and numerical analyses (Figure 10), a temperature increase is observed in the embedded layer around the sleeve when installed without a cap. This is where the surface temperature rises on the exterior wall finish. The reason is higher heat flux at the joint, which causes specific stains on the exterior wall finish (Figure 11a), referred to as the ladybug effect [57,59].

By using the installation method with a Styrofoam plug, you can reduce the temperature disturbance fields around the connector and ensure virtually uniform temperature distribution in the plaster and the embedded plaster layer.

In the first phase of operation, especially in the autumn–winter period, as well as after the precipitation period, in the places of connectors that pierce the thermal insulation material, the so called ‘ladybug effect’ appears. This is a consequence of the favorable drying conditions of the plaster in these areas: increased temperature due to point thermal bridge, hydrophobic anchor plate substrate. Later, these areas are free from biocorrosion. Figure 10 shows different exterior walls of the same building, while Figure 11a presents a fragment of an external wall where anchors completely pierced the insulation layer and a ladybird effect can be observed.

Figure 11b shows a fragment of the newly added part of the facility, which has been in operation since 2016, and biological corrosion can already be observed. However, in this case, anchors were embedded in the insulation layer and the ladybird effect does not occur.

## 4. Conclusions

As seen in the conducted analysis, the problem of microbial growth on exterior wall finish is a complex effect influenced by a number of interacting factors related to the abiotic conditions of the exterior wall finish system (Table 4 and Table 5).

Tests performed on ETICS systems indicate high surface moisture of the plaster coating as the main cause of biocorrosion. The moisture content of plaster results from a combination of two basic groups of factors: plaster properties and external environment.

The thickness of the plaster layer and its microstructure are the decisive parameters that affect the time needed for water to evaporate from the soaked plaster. The thicker the layer, the longer it takes for water to evaporate from the finish layer surface. Predicting biocorrosion risk involves the correlation of these two coefficients.

A natural barrier against corrosion is the high pH of the substrate, which is specific for mineral plasters. However, as time passes, it is observed to be decreased due to plaster hardening and aging. In the long term, and in combination with the plaster properties (hydrophilicity, microstructure), the biocorrosion process is more intense.

Water absorption and retention are determined by the hydrophilicity and porosity of the plaster as well as pore distribution. For cement-based plasters, the porosity structure changes during the composite hardening process. This process is long and can take over a year. This leads to a reduction in capillarity and, consequently, to improved moisture conditions.

In a layered ETICS system, the moisture flux values depend on the adopted material system as well as the internal and external climate. Condensation planes most often occur at the connection of the thermal insulation layer and the reinforcement layer as well as at the junction of the thin-coat plaster and the paint coating. The intensity of this process depends on the plaster composition and microstructure. The ability of the reinforcing layer and the insulation material to dry depends primarily on the plaster diffusivity (possibly with paint). In the transitional seasons, surface condensation during the night period is an important component of the moisture balance. It is a result of low thermal inertia of the plaster and the influence of long-wave radiation from the sky. In this case, the drying process depends on solar radiation and the absorption of short-wave radiation by the exterior wall finish surface.

The drying process on shaded surfaces primarily depends on the outdoor air parameters. It is much less efficient and, as a result, the plaster on north-facing exterior walls remains damp for most of the day. This promotes the contamination deposition, which is a breeding ground for microorganisms. The plaster roughness intensifies the contamination retention in the intergranular areas, thus creating favorable colonization conditions. As seen in the conducted analysis, internal factors that directly affect colonization are widely recognized. The opposite issue, i.e., the influence of microbial growth on plaster properties, is mainly considered in the context of changing the pH of the substrate.

## Figures and Tables

**Table 1:** Determined pH values for plaster based on the fungus type and test date (own table based on [26]).

Material	Fungus Type	Standard SampleNot Contaminated	pH after 1 Month after Contamination	pH after 6 Months after Contamination
Plaster	Aspergillus	12.21	12.00	9.63
	Cladosporium		12.04	10.40
	Penicillium		12.06	10.04

**Table 2:** Porosity changes of mortar samples in the CAT chamber exposed to simulate environment (own table based on [48]).

Type of Mortar		Total Porosity Changes (%)			
		State 0	100 Cycles	200 Cycles	300 Cycles
Cement	sC	8.66	11.46	12.25	17.63
Cement-lime	sCL	10.14	12.38	15.46	19.76
Cement-lime Y	sLY	8.71	11.65	12.21	16.94
Cement E	sCE	8.55	10.14	12.19	16.32
Cement E + Ahydrosil	sCEA	10.76	12.01	14.21	17.46

**Table 3:** Classes of vapor permeability of plasters according to PN-EN 1062-1 (own table based on [58]).

Class	s_d_ [m]	Coating Type
I	<0.14	Mineral, silicone and silicate-organic
II	0.14–1.4	Acrylic with reduced binder content
III	>1.4	Acrylic with high binder content

**Table 4:** Relationship between plaster properties and microbial colonization.

Plasters Properties	Influence of Plaster Properties on Microorganism Colonization		Influence of Microorganisms on Plaster Properties	
	Direct	Indirect	Direct	Indirect
Plaster thermal capacity	(widening of temperature range supporting colonization)	(humidity changes resulting from surface condensation)	not tested	(humidity changes resulting from microstructure changes resulting from freezing and thawing of humid coatings)
Plaster composition	(influence of plaster type on microorganism colonization)	(humidity changes resulting from water absorption)	(in context of chemical changes)	(humidity changes resulting from microstructure changes due to freezing and thawing of humid coatings)
Plaster pH	(influence on microorganisms colonization)	not tested	(substrate pH changes due to microorganisms colonization)	not tested)
Surface structure	(roughness effect)	not tested	not tested	not tested)
Plaster porosity	not tested	(porosity changes due to freeze-thaw cycles and aging)	not tested	(changes resulting from microstructure, changes resulting from freezing and thawing of humid coatings)

**Table 5:** Relationship between solutions for material layouts and building details and microbial colonization.

System Properties	Influence of Solutions Material Layouts and Building Details on Microorganism Colonization		Influence of Microorganisms on Solutions for Material Layouts and Building Details	
	Direct	Indirect	Direct	Indirect
Thermal requirements for envelope components	(widening of temperature range which increases colonization)	(periods of summer condensation, changes in thermal conductivity of the system due to plaster microstructure changes)	not tested	(humidity changes due to microstructure changes resulting from freeze-thaw cycles of humid coatings)
Plaster color scheme	not tested	(stresses in the plaster layers, cyclic material deformation, microcracks)	not tested	not tested
Exterior coating material systems	not tested	(humidity changes due to internal condensation)	not tested	(humiditychanges due to microstructure changes resulting from freezing and thawing of humid coatings)
Structural solutions of ETICS system	(in situ research of influence of anchoring system on biocorrosion development)	(increasing temperature on plaster surface in anchoring surface, changes in dynamics of drying resulting from various plaster substrate)	not tested	not tested

## References

[1] Said M.N., Brown W.C., Walker I.S. (1997). Long-term field monitoring of an EIFS cladwall. J. Build. Phys..

[2] Künzel H., Künzel H.M., Sedlbauer K. (2006). Long-Term Performance of External Thermal Insulation Systems (ETICS). Acta Sci. Pol. Archit..

[3] Kvande T., Bakken N., Bergheim E., Thue J.V., Sedlbauer K. (2018). Durability of ETICS with Rendering in Norway—Experimental and Field Investigations. Buildings.

[4] Duarte C. The Europe of mortars and ETICS. Trends, perspectives and opportunities. Proceedings of the IX SBTA—Brazilian Symposium on Mortars Technology.

[5] Pasker R. Activitatea EAE pentrucalitate a sistemelor ETICS pe plan European. Proceedings of the III Forum ETICS.

[6] (2019). External thermal insulation composite systems (ETICS) with renderings.

[7] (2013). External thermal insulation composite systems (ETICS) with renderings.

[8] Barreira E., De Freitas V. (2014). External Thermal Insulation Composite Systems: Critical Parameters for Surface Hygrothermal Behaviour. Adv. Mater. Sci. Eng..

[9] Johansson S. (2011). Biological Growth on Rendered Facades. Ph.D. Thesis.

[10] Ksit B., Horbik D. (2015). Zanieczyszczenia biologiczne elewacji. Builder.

[11] Johansson S., Wadso L., Sandin K. (2010). Estimation of mould growth levels on rendered façades based on surface relative humidity and surface temperature measurements. Build. Environ..

[12] Krus M., Rösler D., Sedlbauer K. New model for the hygrothermal calculation of condensate on the external building surface. Proceedings of the 3rd International Building Physics Conference, Building Physics and Building Engineering.

[13] Ilomets S., Heim D., Chodak E., Czarny D., Kalamees T. (2020). A method to develop energy activated ETICS. E3S Web of Conferences.

[14] Veiga R. (2004). Pathology and Repair of Several Types of Wall Rendering. Course of Pathology of Walls’ Claddings and Strategies to Avoid Them.

[15] Rokiel M. (2017). Tynki zewnętrzne chroniące przed wilgocią. Mater. Bud..

[16] Wiejak A., Miklaszewska J. (2012). Odporność na glony tynków cienkowarstwowych do stosowania na zewnątrz obiektów. Mater. Bud..

[17] Konca P. (2005). Badania laboratoryjne wybranych wypraw elewacyjnych w systemach ociepleń z zastosowaniem styropianu jako materiału termoizolacyjnego. Fizyka Budowli w Teorii I Praktyce.

[18] Karyś J. (2014). Ochrona Przed Wilgocią I korozją w Budownictwie.

[19] Blaich J. (1999). Aussenwände mit Wärme-Damm-Verbundsystem. Algenund Pilzbewuchs. Deutches Arkit..

[20] Parracha J.L., Borsoi G., Flores-Colen I., Veiga R., Nunes L., Dionisio A., Gloria Gomes M., Faria P. (2021). Performance parameters of ETICS: Correlating water resistance, bio-susceptibility and surface properties. Constr. Build. Mater..

[21] Kozakiewicz J., Ofat I., Trzaskowska J. (2015). Silicone-containing aqueous polymer dispersions with hybrid particle structure. Adv. Colloid Interface Sci..

[22] Wojciechowski K., Kaczorowski M., Mierzejewska J., Parzuchowski P. (2018). Antimicrobial dispersions and films from positively charged styrene and acrylic copolymers. Colloids Surf..

[23] Khanjani J., Hanifpour A., Pazokifard S., Zohuriaan-Mehr M.J. (2020). Waterborne acrylic-styrene/PDMS coatings formulated by different particle sizes of PDMS emulsions for outdoor applications. Prog. Org. Coat..

[24] Stanaszek E. (2004). Procesy bioterioracji materiałów budowlanych. Cem. Wapno Beton.

[25] Pogorzelec P. (2019). Korozja mikrobiologiczna ocieplonych fasad budynkówi jej aktywne zapobieganie. Izolacje.

[26] Stanaszek-Tomal E. (2017). The Problem of Biological Destruction of Façades of Insulated Buildings—Causes and Effects. IOP Conference Series: Materials Science and Engineering.

[27] Horbik D. (2013). Biodeterioracja a Trwałość Elewacji Obiektów Budowlanych o Różnym Przeznaczeniu. Ph.D. Thesis.

[28] Tran T., Govin A., Guyonnet R., Grosseau P., Lors C., Damidot D., Deves O., Ruot B. (2014). Influence of the intrinsic characteristics of mortars on their biofouling by pigmented organisms: Comparison between laboratory and field scale experiments. Int. Biodeterior. Biodegrad..

[29] Barberousse H., Brayner R., Do Rego A.M.B., Castaing J.-C., Beurdeley-Saudou P., Colombet J.-F. (2007). Adhesion of facade coating colonisers, as mediated by physico-chemical properties. Biofouling.

[30] Barberousse H., Ruota B., Yepremianb C., Boulonc G. (2007). An assessment of facade coatings against colonisation by aerial algae and cyanobacteria. Build. Environ..

[31] Becker R. (2003). Patterned staining of rendered facades: Hygrothermal analysis as a means for diagnosis. J. Therm. Envel. Build. Sci..

[32] Sedlbauer K., Krus M. (2002). Mold growth on ETICS (EIFS) as a result of “bad workmanship”?. J. Therm. Envel. Build. Sci..

[33] Venzmer H., Von Werder J., Lesnych N., Koss Krus L. Algal defacement of facade materials—results of long term natural weathering tests obtained by new diagnostic tools. Proceedings of the 8th Symposium on Building Physics in the Nordic Countries.

[34] Barreira E., Freitas V.P. (2013). The effect of nearby obstacles in surface condensations on external thermal insulation composite systems: Experimental and numerical study. J. Build. Phys..

[35] Barreira E., Freitas V.P. (2013). Experimental study of the hygrothermal behaviour of External Thermal Insulation Composite Systems (ETICS). Build. Environ..

[36] Zamorowska R., Nowak B. (2006). Mikrobiologiczne skażenie elewacji budynków. Mater. Bud..

[37] Ryparova P., Tresarek P. (2017). The Dependence of Mould on the Relative Humidity in Different Types of Materials. Key Eng. Mater..

[38] Ryparova P., Tresarek P., Racova Z., Trejbal J. External thermal insulation composite system and problem with biodegradation. Proceedings of the Central Europe towards Sustainable Building 2016: Innovations for Sustainable Future.

[39] Neville A.M. (2000). Properties of Concrete.

[40] Fagerlund G. (1997). Durability of Concrete Structures.

[41] Tada S. (1996). Microstructural approach to frost resistance of highly porous materials. Durab. Build. Mater. Compon..

[42] Zhang B. (1998). Relationship between pore structure and mechanical properties of ordinary concrete under bending fatigue. Cem. Concr. Res..

[43] Kearsley E.P., Wainwright P.J. (2002). The effect of porosity on the strength of foamed concrete. Cem. Concr. Res..

[44] Kumar R., Bhattacharjee B. (2003). Porosity, pore size distribution and strength of concrete. Cem. Concr. Res..

[45] Zhihua P., Dongxu L., Jian Y., Yang N. (2003). Properties and microstructure of the hardened alkali-activated red mud-slag cementitious material. Cem. Concr. Res..

[46] Moropoulou A., Polikreti K., Bakolas A., Michailidis P. (2003). Correlation of physicochemical and mechanical properties of historical mortars and classification by multi variate statistics. Cem. Concr. Res..

[47] Matusinović T., Šipušić J., Vrbos N. (2003). Porosity–strength relation in calcium aluminate cement pastes. Cem. Concr. Res..

[48] Bochen J., Gil S., Szwabowski J. (2005). Influence of ageing process on porosity changes of the external plasters. Cem. Concr. Compos..

[49] Martinez-Ramirez S., Puertas F., Blanco-Varela M. (1997). Studies on degradation of lime mortars in atmospheric simulation chambers. Cem. Concr. Res..

[50] Polish Ministry of Infrastructure (2009). Regulation of the Minister of Infrastructure of 12 April 2002 on the technical requirements to be met by buildings and their location (with later changes). J. Laws.

[51] Rokiel M. (2019). Problemy techniczne stosowania ciemnych tynków. Mater. Bud..

[52] Dylla A. (2009). Practical Thermal Physics of Buildings.

[53] IMGW. https://www.imgw.pl/wydarzenia/charakterystyka-wybranych-elementów-klimatu-w-polsce-w-sierpniu-2021-podsumowanie-sezonu.

[54] Holm A., Zillig W., Kunzel H. Exterior surface temperature and humidity of walls—Comparison of experiment and numerical simulation. Proceedings of the Performance of Exterior Envelopes of Whole Buildings IX.

[55] Zillig W., Lenz K., Krus M., Carmeliet J., Hens H., Vermeir G. (2003). Condensation on facades—Influence of construction type and orientation. Research in Building Physics.

[56] Wesołowska M., Wesołowska M. (2016). Ochrona Murów Licowych Przed Wpływem Środowiska.

[57] Krus M., Fitz C., Holm A. Prevention of algae and mould growth on facades by coatings with lowered long-wave emission. Proceedings of the 3rd International Building Physics Conference—Research in Building Physics and Building Engineering.

[58] EN 1062-1 (1997). Paints and Varnishes—Coating Materials and Coating Systems for Exterior Masonry and Concrete—Part 1: Classification.

[59] Orlik-Kożdoń B., Nowoświat A., Krause P., Ponikiewski T. (2018). A numerical and experimental investigation of temperature field in place of anchors in ETICS system. Constr. Build. Mater..

